# Vitamin C: A Preventative, Therapeutic Agent Against Helicobacter pylori

**DOI:** 10.7759/cureus.3062

**Published:** 2018-07-30

**Authors:** Azhar Hussain, Elsa Tabrez, Jagannadha Peela, Prasanna Honnavar, Shams S.M. Tabrez

**Affiliations:** 1 Medicine, Xavier University School of Medicine, Oranjestad, ABW; 2 Medicine, St. Matthew’s University School of Medicine, George Town, CYM; 3 Faculty of Medicine/Professor of Medical Genetics and Biochemistry, St. Matthew's University School of Medicine, Grand Cayman, CYM; 4 Microbiology and Immunology/Faculty of Medicine, Xavier University School of Medicine, Oranjestad, ABW; 5 Board Certified Gastroenterologist and Hepatologist, University of Central Florida College of Medicine, Orlando, USA

**Keywords:** peptic ulcer disease, vitamin c supplementation, urease, vitamin c, helicobacter pylori infection

## Abstract

The treatment of *Helicobacter pylori* (*H. pylori*) induced infections using antibiotic therapies is clinically well accepted; however, using a noninvasive approach with the implementation of therapeutic agents such as vitamin C is not well investigated. Vitamin C has certain characteristics, which allow for it to be considered as a potential treatment option for patients with *H. pylori* infections. Vitamin C’s hostility and mechanism of action towards *H. pylori *infection in peptic ulcer disease can be classified into two categories: as a preventative agent and alternatively as a therapeutic agent. Preventatively vitamin C acts as a biological antioxidant as well as an immune boosting agent, while therapeutically it acts as an inhibitor of urease, a potential collagen synthesizing agent, and a stimulant in prostaglandin synthesis. As a result, the dosage of vitamin C should be highly regulated. Furthermore, numerous studies have shown that vitamin C supplementation if taken with antibiotics can increase the efficiency of the treatment leading to an increased possibility of eradication of *H. pylori* in infected individuals. This paper will investigate the recent studies that show different mechanisms through which vitamin C can be used as a preventative or a therapeutic agent for the treatment of *H. pylori* related infections.

## Introduction and background

*Helicobacter pylori* (*H. pylori*) is a Gram-negative, microaerophilic, spiral-shaped bacterium that colonizes on the mucosal lining of the stomach [1]. *H. pylori* is one of the primary causes of upper gastrointestinal diseases, including dyspepsia, peptic ulcer diseases, heartburn, and gastroesophageal reflux disease. Chronic disease due to *H. pylori* has been associated with the advancement of gastric adenocarcinoma and lymphoma involving mucosa-associated lymphoid tissue (MALT) [2]. Some 95% of the patients with *H. pylori* infection develop duodenal ulcers, 80% of the patients develop gastric ulcers, and 10%-15% of the patients develop peptic ulcers. The bacteria transmit via oro-oral, oro-fecal, or oro-gastric route. Recent studies showed that over 50% of the global population is infected by *H. pylori *infection in which 1%-3% develop gastric cancer. As a result, the World Health Organization classified *H. pylori* as a group 1 carcinogen [1].

The proton pump inhibitor (PPI) (e.g., omeprazole 20 mg BID, lansoprazole 30 mg BID, or pantoprazole 40 mg QID) with two antibiotics treatment (such as amoxicillin 1000 mg BID and clarithromycin 500 mg BID) is considered as a standard triple therapy and as a first-line treatment option for *H. pylori* infection [3]. The optimal duration of a standard triple therapy is 14 days achieving a *H. pylori* eradication rate of 81.9%, as compared to 7 days triple therapy which attains an eradication rate of only 72.9% [4]. The standard triple therapy is followed by a second-line treatment, which is a quadruple therapy that consists of a PPI or H2 receptor antagonist (e.g., lansoprazole 30 mg BID or ranitidine 150 mg BID) plus bismuth subsalicylate 525 mg QID, metronidazole 250 mg QID, and tetracycline 500 mg QID for additional 10-14 days for 90.4% eradication [3], but the eradication rate of infection is minimal due to antibiotic resistance and compliance. However, several nonantibiotic treatments have been investigated as potential adjuvants for the treatment of *H. pylori*; these include phytomedicines, probiotics, and antioxidants [5]. The vitamin C content in gastric juice has recently pulled in numerous researchers, suggesting that vitamin C might be a protective agent against the *H. pylori* infection especially against the development of gastric cancer [6]. N-nitroso compounds (NOCs) are strong carcinogens and are closely related to food and nutrition [7]. It has been demonstrated that vitamin C is anticarcinogenic because it inhibits the development of N-nitroso mixes (NOCs) in gastric juice [8].

There have been several clinical studies which demonstrated that high *H. pylori* infection rate is related to low vitamin C (ascorbic acid) level in the gastric juice as well as in the serum [9-10]. Nevertheless, many studies demonstrated that a high dose of vitamin C would inhibit the growth and colonization of *H. pylori* and even eradicate them [11-12]. Understanding the mechanism would help to design more clinical studies in more reasonable ways to formulate appropriate anti-*H. pylori* agents.

## Review

Survival of *H. pylori * at low gastric pH 

*Helicobacter pylori* is not an acidophile, but the main reason for its ability to overcome the acidic gastric environment is due to its ability to synthesize a large amount of urease enzyme that catalyzes the hydrolysis of urea to yield ammonia and carbonic acid [13]. The activation of the urease is a key factor in the successful colonization of bacteria into the gastric mucosa because it can allow the bacteria to survive at a very low acidic pH of 2.5 [1]. However, in the absence of the enzyme urease, the bacteria can only survive at a pH of 4.0-8.0 [13]. Autolysis of the *H. pylori* colony results in the release of cystolic urease into the gastric mucosa, which attaches to the surface of the *H. pylori *bacteria [5, 14]. In the gastric mucosa, the deprotonated carbonic acid and protonated ammonia are in equilibrium [15]. The effect of this reaction is an increase in pH and formation of a basic ammonium cloud around the bacteria allowing *H. pylori* to survive and to colonize on the gastric epithelium [15]. On successful colonization, *H. pylori* resides below the gastric mucus which has a higher pH than the gastric lumen [16].

The motility of the *H. pylori* plays an important role in the pathogenesis and successful colonization into gastric mucosa. For this purpose, *H. pylori* has two to six polar sheathed flagellae, which allow the movement of the bacterium into the highly viscous mucus layer of the gastric epithelium. These flagellae are composed of three main structures: the basal body, which serves as a cell anchor and contains the proteins required for rotation and chemotaxis, a curved hook, and the helically shaped flagellar filament [17]. The lining of the stomach is a spongy gel-like state because of the acid content which the bacterium is unable to penetrate. However, by using its flagella, *H. pylori *releases an adhesion molecule, which allows the bacterium to bind to the host cell [18]. The bacterium then releases a high amount of urease enzyme, which can neutralize the acid by converting urea into carbon dioxide, and ammonia, and drills in the mucoid lining of the gastric epithelium [16].

Discussion: mechanisms of action of vitamin C against *H. pylori*

Vitamin C can be used as a preventative agent as summarized in Table 1 and as a therapeutic agent as summarized in Table 2. This review focuses on the mechanism through which vitamin C can be preventative: as beneficially used to prevent *H. pylori* infection as well as therapeutic: as to control the infection and eradicate the bacteria.

              I.         Vitamin C as a Preventative Agent 

**Table 1:** Role of vitamin C as a preventative agent.

Preventative agent	
Role	Function
Biological antioxidant	Ascorbic acid scavenges and eliminates free radicals
Immune booster	100-fold increase of vitamin C inside immune cells and decrease of plasma vitamin C

*Biological Antioxidant* 

Vitamin C is a nonessential, potent, water-soluble micronutrient that can neutralize a wide range of pro-oxidants, due to its low redox potential [19-20]. Vitamin C functions as a biological antioxidant, an oxidative stress reducer, a factor in immune function and in enzyme activation as shown in Table 1 [19]. Vitamin C also acts as a cofactor in the biosynthesis of collagen, catecholamines, and peptide hormones [21]. Vitamin C exists in two major forms: reduced form as ascorbic acid, as well as its oxidized form as dehydroascorbic acid, which may be interconvertible [20], by a dehydroascorbic acid reductase, glutaredoxins or other thiols acting as an electron donor [5]. The reduced form as ascorbic acid has scavenger properties and may be beneficial to eliminate free radicals under the formation of semidehydroascorbic acid, which is a nonreactive radical [22]. The dehydroascorbic acid may spontaneously hydrolyze and dehydrate; however, the ascorbic acid is more stable and does not show the same tendency to irreversibly hydrolyze particularly at pH > 4 [5]. This mechanism is essential for the inhibition of the growth of *H. pylori *[16, 18, 23].

*Immune Boosting Agent* 

The immune system within the human body acts as a protective agent against pathogens that cause infections and diseases. These immune responses are divided into two categories; an innate system is the first nonspecific immune response, while the adaptive immune response is pathogen-specific and develops over time subsequent to the introduction of that particular pathogen [23-24]. The innate immune response is particularly very important in children because it exists at birth and offers initial protection against foreign pathogens. Adaptive immune responses are different in that they are dependent upon prior exposure to the antigens. The adaptive immune response, therefore, has mature plasma B cells and antibodies, which are capable of recognizing specific previously encountered antigens. Each successful immune response concludes with phagocytic engulfing of pathogens by macrophages [24].

One of the most important functions of vitamin C is that it helps in the activation of the immune system of the body. Within the plasma membrane of the immune cells, there are active transporters of vitamin C that bind to it and actively transport the vitamin C into the cell [25]. For example, during inflammation due to infection, these transporters increase the influx of vitamin C up to 100-fold compared to the amount of vitamin C present in the plasma [26]. As a result, plasma vitamin C concentration can be depleted during infection. Studies show that the aging of the immune system can be reversed by the supplementation of vitamin C [27]. This study shows significant results in geriatric patients whose overall immune function is in a process of degradation. 

II.            Vitamin C as a Therapeutic Agent 

**Table 2:** Role of vitamin C as a therapeutic agent.

Therapeutic agent	
Function	Mechanism of action
Urease maturation (potent virulence factor required for survival of *H. pylori* in acidic environments)	Increase in vitamin C leads to the reduction of nickel of urease enzyme
Collagen synthesis	Vitamin C acts as a cofactor for synthesizing collagen type IV required for synthesis of lamina propria in the stomach lining. The absence of vitamin C allows easy penetration of *H. pylori*
Prostaglandin synthesis	Phospholipid molecule converts to arachidonic acid via the enzyme phospholipase A2. Arachidonic acid is then converted to prostaglandin via the enzymes cyclooxygenase 1 (COX1) and cyclooxygenase 2 (COX2)

Inactivation of Urease

**Figure 1:**
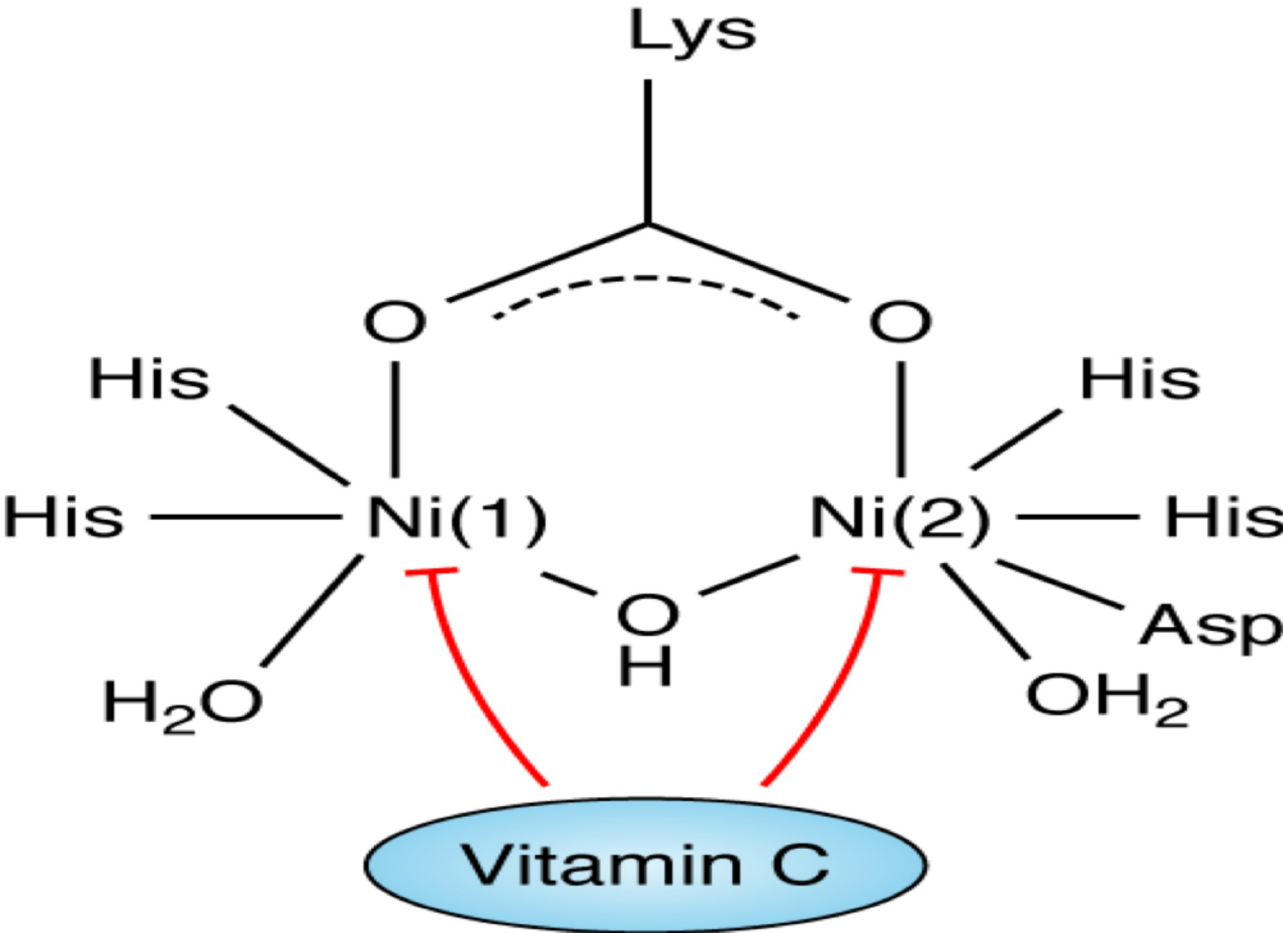
The structure of urease and the role of vitamin C as an inhibitor.

Urease is an important enzyme that constitutes approximately 5%-6% of the total protein of H. pylori and it is linked to its pathogenicity as shown in Table 2 [28], due to its ability to colonize on the gastric mucosa at a low pH [23]. The structure of urease enzyme is composed of two half subunits held together by a noncovalent bond [5], each subunit containing a specific active site. These active sites contain two nickel ions that are bound by a carbamylated lysine and an oxygen donor [29]. In addition to the binding, the first nickel ion is held by two histidine amino acids and a water molecule and the second nickel ion which is similar in composition to the first nickel ion, also contains the amino acid aspartate as shown in Figure 1 [5, 14, 28-29]. The ability of vitamin C to inhibit urease action plays an important role in understanding the mechanism of *H. pylori* infection and bacterial eradication [30]. Studies show that the high concentration of vitamin C favors reduction of the nickel center in the urease enzyme [5], which in turn inhibits the activity of the enzyme and may reduce the *H. pylori* manifestations.

Vitamin C, which is a relatively strong acid (pKa = 4.1) further lowers the pH of the gastric lumen [28]. Vitamin C, a reducing agent of urease, when added to the gastric lumen results in urease becoming structurally unstable, therefore, irreversibly losing its enzyme activity [30]. Therefore, vitamin C can be beneficial in inhibiting the growth, colonization, and endurance of *H. pylori *at an earlier period of the infection and may be helpful in the eradication of the bacteria [23].

Collagen Synthesizing Agent

**Figure 2:**
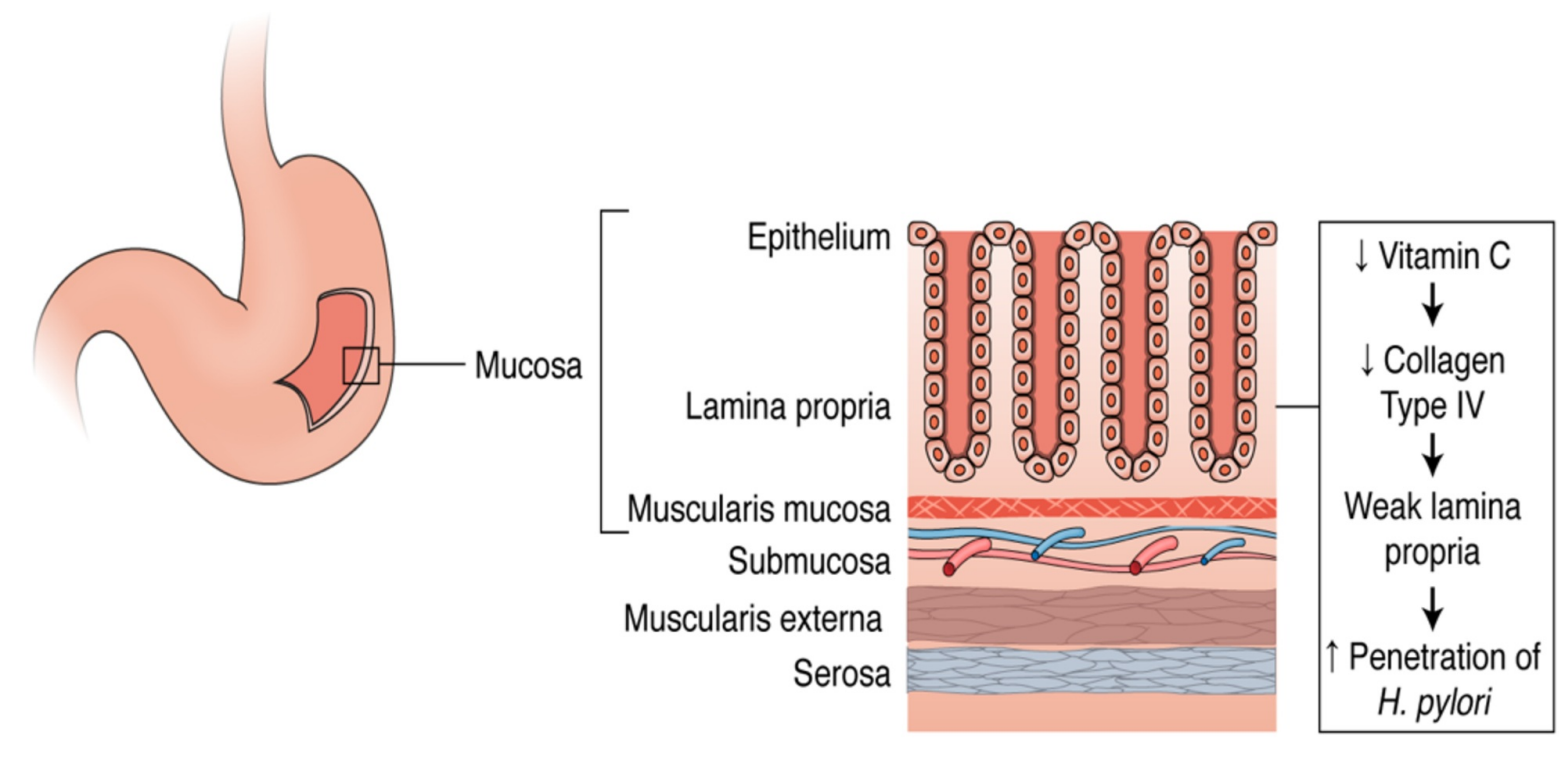
Role of vitamin C in collagen synthesis.

The stomach inner lining itself is a layered structure containing four sublayers which are the mucosa, the submucosa, the muscular, and the serosa. The mucosa layer of the inner stomach lining is composed of epithelial cells specific to the stomach including parietal cells, chief cells, and gastric enteroendocrine cells. Below this layer of epithelial cells in the mucosa is the lamina propria. Under the mucosa layer is the submucosa which entirely contains blood vessels. *H. pylori* can bind to the extracellular matrix (ECM) proteins on the surface of the epithelial cells and infiltrate the cells by releasing toxins as shown in Figure 2 [31]. Once H. pylori infiltrates the cells, it can further penetrate the deeper layers of the stomach lining and travel through the bloodstream. Presuming that *H. pylori *can only penetrate farther into tissues if it passes through the lamina propria, a durable lamina propria has the potential to prevent penetration. The lamina propria is composed predominantly of collagen fibers, specifically collagen type IV which provides structure and support for the epithelial cells located within the gastric mucosa [32]. Vitamin C is a known cofactor in the synthesis and strengthening of collagen [33]. Vitamin C's collagen strengthening abilities could potentially be a significant reason as to why patients with an increased serum and plasma vitamin C experience very little to no infestation of *H. pylori* [34]. Stronger collagen in the lamina propria under the epithelium could attribute to a more difficult infiltration mechanism of *H. pylori *into the tissues and bloodstream, therefore, resulting in decreased penetration and decreased overall prevalence of *H. pylori* linked diseases [33].

Vitamin C is specifically required for hydroxylation in collagen synthesis, a post-translational modification [35]. Once pre-procollagen is translated from mRNA, it enters the rough endoplasmic reticulum for elongation, hydroxylation, and glycosylation prior to its maturation. Hydroxylation of pre-procollagen allows for further glycosylation of the molecule and subsequent triple helix formation. Both enzymes, lysyl hydroxylase and prolyl hydroxylase are necessary for hydroxylation and require vitamin C as a cofactor amongst other elements such as O_2_, ferrous (Fe^2+^), and alpha-ketoglutarate [34]. Without vitamin C, the enzymes are not able to function and additional modification of pre-procollagen does not take place, in which case the molecule does not mature to collagen [35]. An increase of vitamin C in the plasma of an individual could account for an increase in collagen synthesis, therefore, resulting in a tougher collagen-saturated lamina propria [32]. This relationship has the potential for being a possible correlating factor between the high vitamin C serum and plasma concentrations and low occurrence of *H. pylori* penetrance in certain individuals.

*Prostaglandin Synthesis* 

Prostaglandins (PG) are lipid-derived compounds, subdivided into PGA, PGB, PGE, and PGF [36], which have inflammatory responses and hormone-like effects on various reactions within the body [37]. The effect of specific prostaglandins is dependent on its location of the action. Prostaglandin E2 (PGE2) is found in high concentrations within the gastric juice and mucosa, and thus has forceful protective effects on the gastric mucosa layer of the inner stomach lining [38]. PGE2s are protective in the sense that they stimulate mucosal blood flow as well as mucus and bicarbonate secretion within the lumen of the stomach. The release and activation of PGE2s are contingent upon injury to the mucosal layer [39]. Subsequent to *H. pylori* penetration of the epithelial cells within the mucosa [37], PGE2s are released to secrete mucus and act as defensive agents [39]. A high degree of prostaglandin synthesis is most likely related to a high defense against bacterial agents like *H. pylori* [38].

Certain studies have identified a correlation between vitamin C and the synthesis of prostaglandin E2 specifically [40]. A study was conducted using inbred mice to assess the influence of vitamin C on prostaglandin E2 synthesis. A 90%-100% increase in PGE2 output was noted upon the introduction of vitamin C [41]. Prostaglandin synthesis begins with the conversion of a phospholipid molecule to arachidonic acid in the presence of the enzyme phospholipase A2. Arachidonic acid undergoes a cyclooxygenase reaction and a peroxidase reaction in the presence of enzymes COX-1 and COX-2, to produce prostaglandin which becomes tissue-specific depending upon location [42]. El Attar et al. showed that vitamin C has dose-dependent effects on the release of arachidonic acid which leads to further PGE2 synthesis. In addition to that, vitamin C acts as a stimulant of PGE2 synthesis by inducing the release of exogenous arachidonic acid in fibroblast cells [40]. Similar findings observed by Siegel et al. show an abundance of mucous production upon abrasion of the mucosal layer from *H. pylori* [41].

Dose regulation of vitamin C

Vitamin C is a water-soluble vitamin and an essential nutrient that must be taken from the diet. The optimum dietary vitamin C intake of 200 mg per day is essential to increase vitamin C’s health benefits with the least risk of adverse effects in the majority of the adult population [43]. Normally an excess dose of a water-soluble vitamin simply passes through the body without causing any toxic effects; however, if vitamin C consumption is more than 2000 mg per day, it can cause kidney stones as well as osmotic diarrhea due to limited absorption [44].

Recently there have been numerous studies that discuss the dosage of vitamin C in the treatment of *H. pylori*.  Zojaji et al. conducted a study in which two groups were given amoxicillin 1 g with metronidazole 500 mg BID and bismuth 240 mg BID with omeprazole 40 mg QID in two divided doses. The second group was given an additional 500 mg of vitamin C. Experimental results showed that 78% of individuals of group two with the additional vitamin C were able to eradicate *H. pylori* as compared to 48.8% of individuals from group one [45].

Similar results were shown byJarosz et al. in which two groups of patients with *H. pylori* infection were treated without the administration of any antibiotics. The control group was treated with antacids for four weeks whereas the second group was treated with the same antacids for four weeks with an additional dose of 5 g of vitamin C daily for a span of four weeks. Plasma and gastric juice total vitamin C levels were measured at baseline, at the end of four weeks’ treatment, and again four weeks after treatment cessation. In the control group, *H. pylori* infection remained unchanged in all the patients; however, the patients with vitamin C treatment were able to eradicate the *H. pylori* (p = 0.01) [46]. These studies do show promising results; however, more research must be conducted to determine the best treatment options accompanied by vitamin C dosages for the treatment of *H. pylori*.

Additional clinical trials

Table 3 shows the clinical trials that have been previously conducted to assess the effectiveness of vitamin C with or without the use of antibiotics. These clinical trials further suggest that vitamin C may be beneficial for decreasing the incidence rate for *H. pylori* related infections.

**Table 3:** Clinical trial data analysis.

Author	Publication date	Patients	Treatment	Results
Sezikli et al. [47]	April 2012	30 patients with severe gastritis (H. pylori related infection)	Vitamin C 500 mg BID and vitamin E 200 IU BID for four weeks orally	Increase of eradication of H. pylori
Zojaji et al. [45]	September 2009	312 patients with H. pylori infection	Group A:162 patients received amoxicillin 1 g, metronidazole 500 mg BID, bismuth 240 mg BID, and omeprazole 40 QID in two doses Group B: 150 patients received the same regimen plus 500 mg vitamin C	48.8% of the patients in Group A and 78% in Group B responded to eradication therapy
Sasazuki et al. [48]	April 2003	635 patients diagnosed with chronic gastritis (H. pylori related infection), but only 244 finished the treatment	120 patients given low-dose vitamin C (50 mg) and 124 patients given high-dose vitamin C (500 mg) completing five-year supplementation	H. pylori titer was significantly reduced by both low-dose and high-dose vitamin C
Jarosz et al. [46]	December 1998	60 patients with dyspeptic symptoms and proven chronic gastritis, and H. pylori infection Group 1: 28 patients Group 2: 32 patients	Group 1 was treated with antacid for four weeks whereas Group B was treated with antacids for four weeks with an addition of 5 g of vitamin C for four weeks, but no antibiotic treatment in both groups	In the Group A, H. pylori infection remained unchanged in all patients. In Group B, the eradication of H. pylori was 30%.
Chuang et al. [49]	October 2002	104 patients with H. pylori infection	Group 1 was treated with lansoprazole, amoxicillin, and metronidazole BID for a week. Group 2 was treated with lansoprazole, amoxicillin, and metronidazole plus vitamin C (250 mg) and vitamin E (200 mg) BID for a week, followed by vitamin C and vitamin E QD for six consecutive weeks	Vitamin C and vitamin E in combination with triple therapy is not effective and showed no H. pylori eradication
Chuang et al. [50]	January–February 2007	171 H. pylorii nfected patients	Group 1: 55 patients received 20 mg omeprazole, 1 g amoxicillin, and 250 mg clarithromycin BID Group 2: 61 patients received 20 mg omeprazole, 1 g amoxicillin, and 250 mg clarithromycin with additional 500 mg vitamin C BID Group 3: 55 patients 20 mg omeprazole, 1 g amoxicillin, and 500 mg clarithromycin	Group 2 had a higher eradication rate than Group 1 but had an equivalent rate to Group 3. Results indicate that an addition of vitamin C to one week triple therapy can allow for a reduction of the dosage of clarithromycin

However, there are certain studies, as shown in Table 3, that show that vitamin C, if added with the triple therapy regimen, may not improve the *H. pylori* eradication rate. The studies’ inability to elicit a positive correlation between vitamin C and *H. pylori* eradication could be attributed to a particularly low dose [49], a short duration time of the therapy, or the patients’ noncompliance to vitamin C treatment protocols. Further research investigation is needed for the proper protocol through which vitamin C can be added to the triple therapy regimen to increase the eradication of the *H. pylori *infection. These studies must focus on determining the optimum vitamin C dosage and treatment duration required for *H. pylori* eradication.

## Conclusions

When assessing treatment mechanisms with regard to *H. pylori* infections, vitamin C’s role as a potential preventative and therapeutic agent is distinctive. High serum vitamin C levels are associated with a low incidence rate of *H. pylori* infection upon exposure. Vitamin C properties such as biological antioxidant and immune regulator act as a preventative agent against *H. pylori *related infections. Vitamin C also acts as a therapeutic agent by functioning as an inhibitor of urease, a synthesizing agent for collagen, and a stimulant in prostaglandin synthesis. Therefore, while vitamin C’s preventative and therapeutic capacity is significantly under-investigated, current studies have established a distinct positive correlation between vitamin C levels and the body’s ability to combat *H. pylori* infections.
